# Nitrilases NIT1/2/3 Positively Regulate Resistance to *Pseudomonas syringae* pv. *tomato* DC3000 Through Glucosinolate Metabolism in Arabidopsis

**DOI:** 10.3390/ijms252312895

**Published:** 2024-11-30

**Authors:** Shuang Yang, Tianqi Zhang, Pei Yao, Rui Li, Jing Li

**Affiliations:** College of Life Sciences, Northeast Agricultural University, Changjiang Road, Xiangfang District, Harbin 150038, China; yangshuang@neau.edu.cn (S.Y.); 15545336686@163.com (T.Z.); yaopei1016@163.com (P.Y.)

**Keywords:** Arabidopsis nitrilases, glucosinolates, plant disease resistance, plant–pathogen interactions

## Abstract

Nitrilases, found to have a common presence in the plant kingdom, are capable of converting nitriles into their corresponding carboxylic acids through hydrolysis. In Arabidopsis, the nitrilases NIT1, NIT2, and NIT3 catalyze the formation of indole-3-acetonitrile (IAN) into indole-3-acetic acid (IAA). Notably, IAN can originate from the breakdown products of indole glucosinolates. Glucosinolates, which are plant secondary metabolites commonly found in cruciferous plants, and their breakdown products, are crucial for plant defense against pathogens. In our study, we found that nitrilases positively regulate resistance to *Pseudomonas syringae* pv. *tomato* DC3000 (*Pst*DC3000) in mature Arabidopsis. Transcriptome data showed that after *Pst*DC3000 treatment, genes related to the auxin pathway in *nit1nit2nit3* changed more dramatically than in the wild type. Moreover, the enhancement of disease resistance through exogenous aliphatic glucosinolate application relies on NIT1/2/3. Hence, it is hypothesized that NIT1/2/3 may serve a dual role in disease resistance and defense mechanisms. After infection with *Pst*DC3000, NIT1/2/3 catalyzes the biosynthesis of auxin, thereby triggering certain disease-related responses. On the other hand, NIT1/2/3 can also break down nitriles generated from aliphatic glucosinolate degradation to enhance disease resistance. Our study elucidates the regulatory mechanism of nitrilases in Arabidopsis disease resistance, offering a theoretical foundation for enhancing disease resistance in cruciferous plants.

## 1. Introduction

Glucosinolates (GSLs), a distinctive secondary metabolite found in the Brassicaceae family, are prevalent in Arabidopsis and various cruciferous vegetables. They can be categorized into three groups based on the amino acid source: indole glucosinolates from tryptophan, aliphatic glucosinolates from methionine, alanine, and valine, and aromatic glucosinolates from phenylalanine and tyrosine [1,2]. GSLs lack inherent biological activity and are enzymatically degraded by myrosinase through the cleavage of sulfur glucoside bonds, resulting in the liberation of various biologically active breakdown products [3]. Some of these degradation products possess inherent antibacterial properties and demonstrate potent toxicity against pathogens. Additionally, they function as signaling molecules that trigger various defense responses in plants, including the deposition of cell wall callose and the induction of programmed cell death [4,5].

GSLs can undergo degradation into nitrile compounds when myrosinase and nitrile-specific protein (NSP) are present [6,7]. The exogenous introduction of 3-butenitrile (3BN) sourced from glucosinolate has been proven to induce stomatal closure, regulate reactive oxygen species production, and stimulate defense responses, thereby enhancing Arabidopsis resistance to necrotic pathogens [8,9]. Moreover, the biological implications of nitrile compounds remain incompletely understood.

Nitrilases, which are able to convert nitriles into carboxylic acids, were first identified in barley leaves [10]. Nitrilases exhibit broad distribution throughout the plant kingdom, with most of them displaying a diverse array of catalytic substrates and varying significantly in terms of substrate specificity [11,12,13]. Genome sequencing of Arabidopsis revealed the presence of four nitrilase-encoding genes, designated as *NIT1*, *NIT2*, *NIT3*, and *NIT4*. Notably, *NIT1*, *NIT2*, and *NIT3* exhibited sequence similarities exceeding 80%, with NIT1, NIT2, and NIT3 sharing comparable enzymatic properties as isoenzymes [14].

The Arabidopsis nitrilases NIT1, NIT2, and NIT3 play a vital role in accepting indole-3-acetonitrile (IAN) and transforming it into the primary auxin, indole-3-acetic acid (IAA). IAA is a pivotal plant growth regulator that governs growth and development and acts as a defense regulator by modulating plant defense responses through diverse pathways including auxin biosynthesis, signaling pathways, and transport [15]. The impact of auxin on various pathogen types varies significantly. Activation of the auxin signaling pathway can enhance plant resistance against necrotrophic pathogens [16]. Conversely, for biotrophic and hemibiotrophic pathogens such as *Pseudomonas syringae*, *Magnaporthe oryzae*, and *Phytophthora nicotianae*, auxin signaling pathway activation tends to facilitate pathogen invasion. Augmenting auxin signal transduction directly promotes pathogen invasion and colonization within plants [17,18,19].

Nitrilases are pivotal in mediating the relationship between plants and microorganisms. Upon infection with clubroot disease, Arabidopsis exhibits increased expression of *NIT1* and *NIT2* in its roots [20]. Following exposure to *Hyaloperonospora arabidopsidis*, the *nit1*, *nit2*, and *nit3* mutants demonstrated increased susceptibility to disease when compared to the wild type, although the specific mechanism has not been elucidated [21]. In addition, the regulation of nitrilase activity in plants appears to be intricately linked to distinct tissue conditions and developmental stages. The expression of *NIT1/2/3* fluctuates across various stages, with a notable rise in the expression level of *NIT2* observed during leaf senescence [22]. However, *NIT3* is almost not expressed in mature plants, and sulfur-deficient conditions induce its expression [23].

The Arabidopsis nitrilases NIT1, NIT2, and NIT3 are known to play pivotal roles in responding to various adverse environmental conditions, thereby influencing plant growth and development. However, their specific involvement in disease resistance remains understudied, with the underlying mechanisms yet to be elucidated. This study focused on investigating the disease resistance and molecular mechanisms mediated by NIT1, NIT2, and NIT3 using the hemibiotrophic pathogen *Pseudomonas syringae* pv. *tomato* DC3000 (*Pst*DC3000). A comprehensive understanding of the disease resistance mechanisms of these proteins can establish a solid theoretical foundation for enhancing plant immunity against pathogens.

## 2. Results

### 2.1. PstDC300 Induced NIT1/2/3 Expression in Mature Arabidopsis

**Figure 1:**
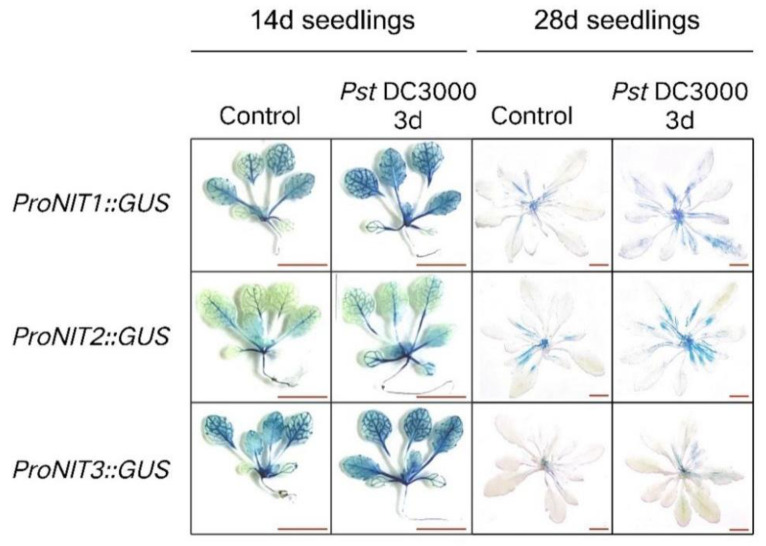
The response of NIT1/2/3 to the injection of PstDC3000 in 14-day-old and 28-day-old Arabidopsis plants. The spatial expression pattern of GUS under the regulation of the NIT1/2/3 promoter. Bar = 1 cm.

Resistance to *Pst*DC300 has been observed to increase as plants progress in maturity [24]. We investigated the activation of *NIT1/2/3* by *Pst*DC3000 in young (14-day-old) and mature (28-day-old) Arabidopsis plants, respectively. As shown in Figure 1, there was no notable difference in the expression level of *NIT1/2/3* in young seedlings following *Pst*DC3000 treatment. However, the expression level of *NIT1/2/3* was increased in mature seedlings following the same treatment. Moreover, 28-day-old Arabidopsis thaliana (28d seedlings) showed deeper staining of leaf veins after *Pst*DC300 treatment, indicating an increase in *NIT1/2/3* expression, especially NIT3, which is almost not expressed in the mature stage, but gene expression can be observed in the leaf veins after *Pst*DC300 treatment (Figure 1). These results suggest varying responses of *NIT1/2/3* to *Pst*DC3000 at distinct developmental stages in Arabidopsis.

### 2.2. NIT1/2/3 Impacts the Localization of Auxin in Mature Arabidopsis Plants

**Figure 2:**
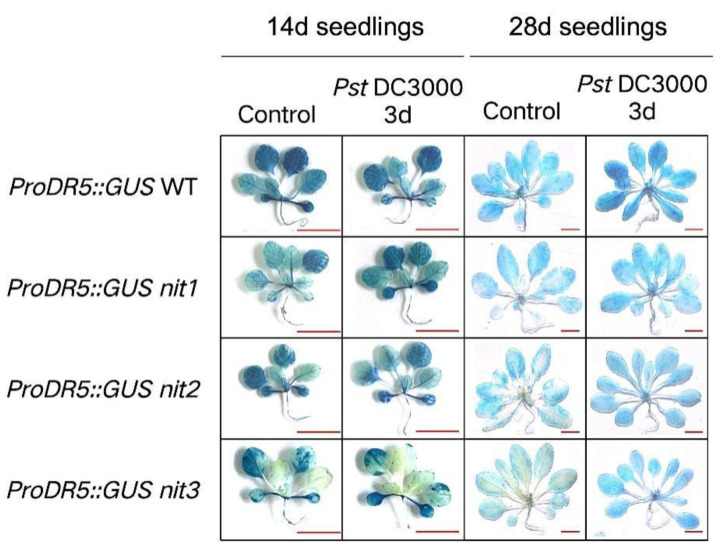
The auxin distribution in 14-day-old and 28-day-old Arabidopsis plants. *Pst*DC3000 infects *ProDR5::GUS* WT, *ProDR5::GUS nit1*, *ProDR5::GUS nit2*, and *ProDR5::GUS nit*3 for a duration of 3 days. Bar = 1 cm.

The DR5 sequence includes seven AuxRE repeats that have the ability to bind to auxin response factors [25]. Through fusion with the GUS reporter gene, the spatial pattern of auxin can be visually observed. The pilot study involved the creation of *ProDR5::GUS* WT, *ProDR5::GUS nit1*, *ProDR5::GUS nit2*, and *ProDR5::GUS nit3* constructs to enable the direct detection of auxin distribution [26]. As shown in Figure 2, there was no significant alteration in auxin levels pre- and post-treatment in 14-day-old seedlings. However, in 28-day-old seedlings exposed to *Pst*DC3000, there was an increase in auxin levels in the WT, while the distribution of auxin was notably altered in the deletion mutant. Prior to *Pst*DC3000 treatment, auxin was predominantly localized at the leaf margin, with a lower presence in the vascular bundle of the mutant, indicating an uneven distribution. After *Pst*DC3000 treatment, the auxin distribution in the mutant became evenly distributed, as observed in the WT (Figure 2). This finding suggests a potential association between NIT1/2/3 and Arabidopsis resistance through the modulation of auxin distribution, although the precise mechanism necessitates further investigation.

### 2.3. NIT1/2/3 Positively Regulates Resistance to PstDC300 in Mature Arabidopsis Plants

**Figure 3:**
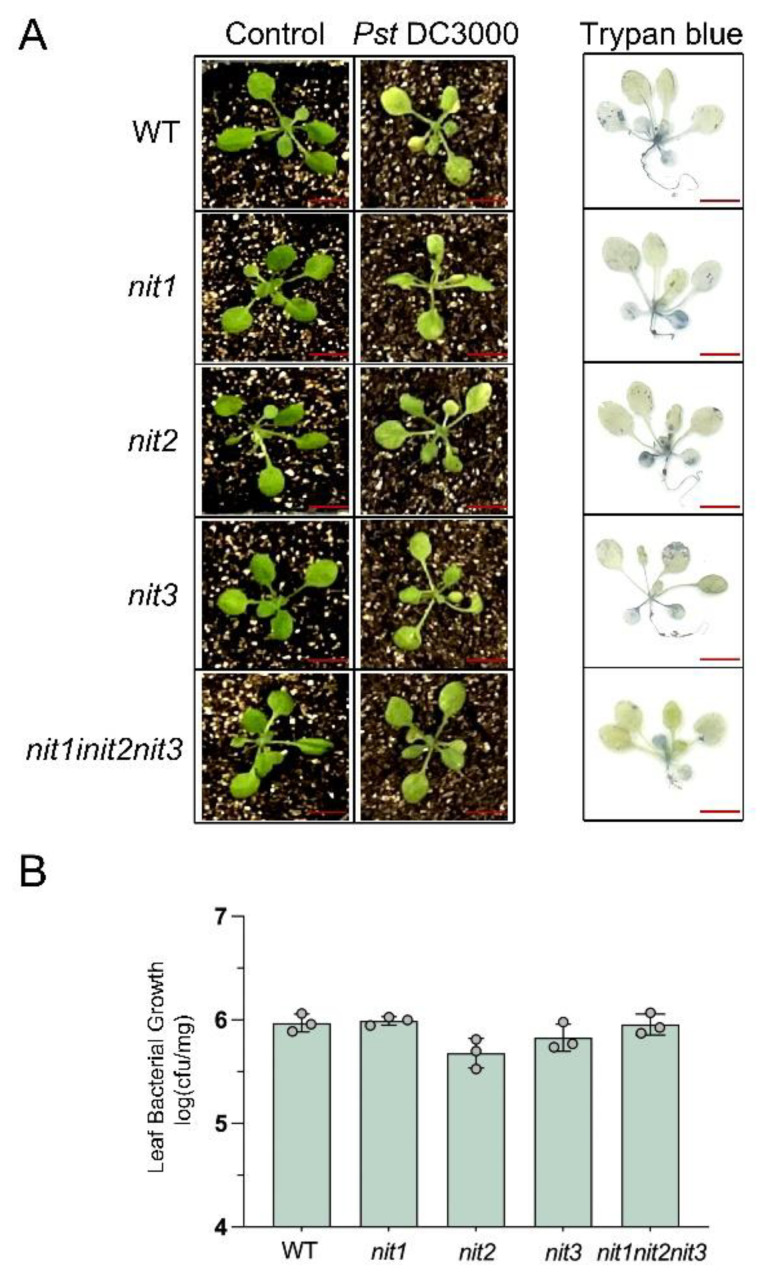
The detection of disease resistance in 14-day-old WT, *nit1*, *nit2*, *nit3,* and *nit1nit2nit3* plants. (**A**) The plants were infiltrated with either a mock treatment or *Pst*DC3000 and subsequently photographed at 3 days post-injection (dpi). The extent of cell death in *Pst*DC3000-treated leaves was assessed using trypan blue staining at 3 dpi. Bar = 1 cm. (**B**) Bacterial growth was assessed in the leaves of 14-day-old WT, *nit1*, *nit2*, *nit3*, and *nit1nit2nit3* plants following treatment with *Pst*DC3000 at 3 dpi. Each sample consisted of leaves from three plants, with three biological replicates collected for each treatment (dots represent individual biological replicates).

**Figure 4:**
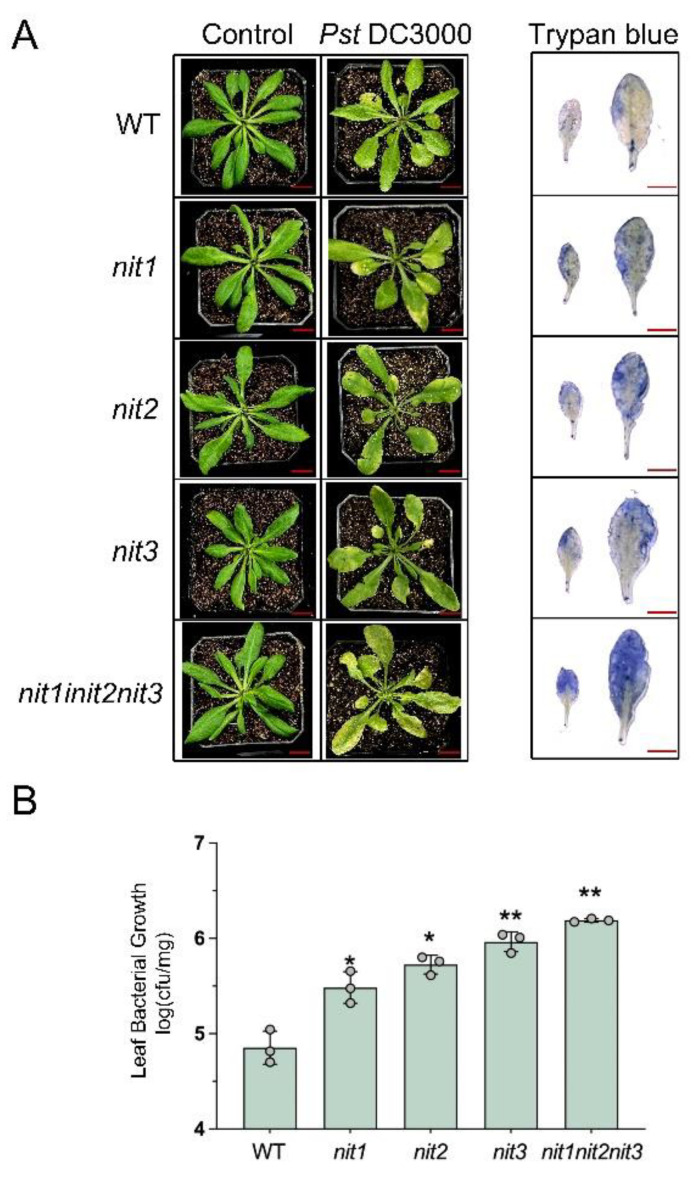
The detection of disease resistance in 28-day-old WT, *nit1*, *nit2*, *nit3,* and *nit1nit2nit3* plants. (**A**) The plants were infiltrated with either a mock treatment or *Pst*DC3000 and subsequently photographed at 3 days post-injection (dpi). The extent of cell death in *Pst*DC3000-treated leaves was assessed using trypan blue staining at 3 dpi. Bar = 1 cm. (**B**) Bacterial growth was assessed in the leaves of 28-day-old WT, *nit1*, *nit2*, *nit3*, and *nit1nit2nit3* plants following treatment with *Pst*DC3000 at 3 dpi. Each sample consisted of leaves from three plants, with three biological replicates collected for each treatment (dots represent individual biological replicates). The asterisks positioned above the bar represent statistically significant variances between *nit1*, *nit2*, *nit3*, *nit1nit2nit3*, and WT, according to *p* < 0.05 (*) or *p* < 0.01 (**) via Student’s *t*-test.

To investigate the impact of deleting the *NIT1/2/3* genes on disease resistance to *Pst*DC3000, both young and mature seedlings of WT, *nit1*, *nit2*, *nit3,* and *nit1nit2nit3* were treated with *Pst*DC3000. The results revealed that 14-day-old seedlings of WT, *nit1*, *nit2*, *nit3*, and *nit1nit2nit3* did not show any notable differences in resistance to *Pst*DC3000 (Figure 3). Following the infection of plants with *Pst*DC3000, cell death was visually observed three days later using trypan blue staining. The observed outcome aligns with the resistant phenotype, showing no significant variances among different genotypes (Figure 3A). Furthermore, there was no significant alteration in the growth of *Pst*DC3000 (Figure 3B). However, 28-day-old WT seedlings displayed higher resistance to *Pst*DC3000 in comparison to 28-day-old *nit1*, *nit2*, *nit3*, and *nit1nit2nit3* seedlings (Figure 4). In comparison to the WT, the deletion mutants exhibited a broader spectrum of yellowing and wilting in their leaves. Among the mutants, *nit1nit2nit3* displayed more pronounced yellowing and wilting symptoms. Trypan blue staining further confirmed a wider blue range in the deletion mutants than in the WT. Among the mutants, *nit1nit2nit3* exhibited the most severe damage, and the blue range of trypan blue staining was the largest (Figure 4A). Additionally, bacterial growth was notably higher in *nit1*, *nit2*, *nit3,* and *nit1nit2nit3* compared to the WT. Notably, *nit1nit2nit3* showed a twenty-fold increase in bacterial quantification compared to the WT (Figure 4B).

### 2.4. NIT1/2/3-Mediated Resistance to PstDC3000 Is Independent of Auxin

**Figure 5:**
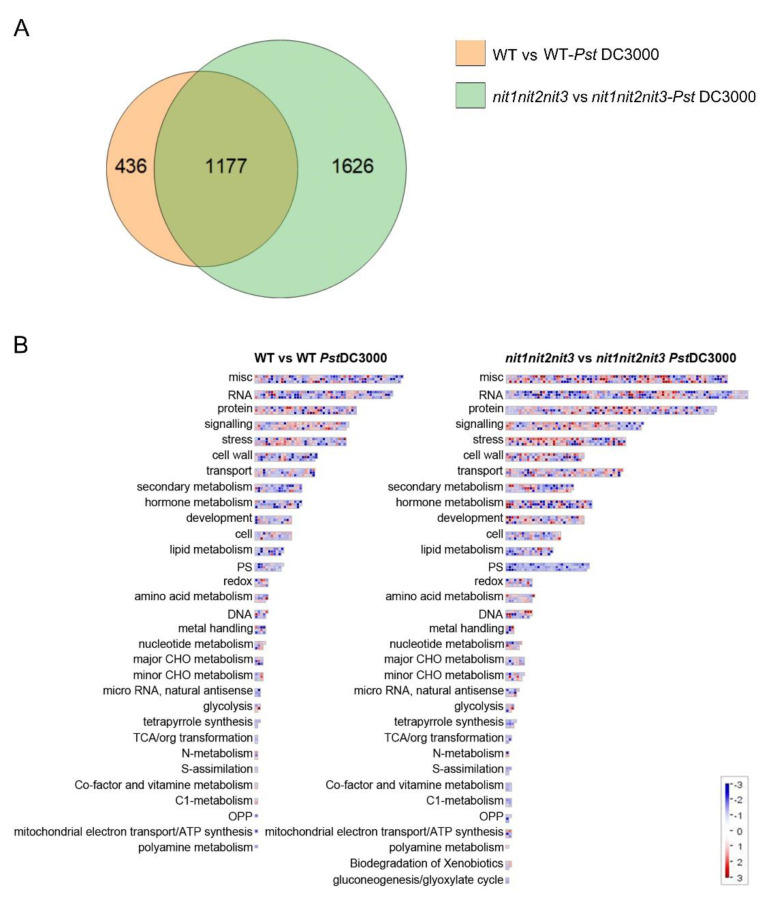
Analysis of transcriptome data from 28-day-old WT and *nit1nit2nit3* plants treated with *Pst*DC3000. (**A**) Differential gene Venn map of the WT before and after *Pst*DC3000 treatment (WT vs. WT-*Pst*DC3000) and *nit1nit2nit3* before and after *Pst*DC3000 treatment (*nit1nit2nit3* vs. *nit1nit2nit3*-*Pst*DC3000). (**B**) Aggregation analysis of differentially expressed genes.

In order to elucidate the molecular mechanisms responsible for the resistance to *Pst*DC3000 mediated by NIT1/2/3, we conducted RNA-seq analyses on 28-day-old WT plants and *nit1nit2nit3* mutants treated with either mock (10 mM MgCl_2_) or *Pst*DC3000 treatments. In response to *Pst*DC3000 treatment, the WT exhibited 1613 differentially expressed genes compared to the mock, while *nit1nit2nit3* mutants showed 2803 differentially expressed genes. Among these differentially expressed genes, 1177 were identical (Figure 5A and Figure S1). Enrichment analysis of the functional classification of differentially expressed genes indicated that both the WT and *nit1nit2nit3* strains exhibited significant enrichment of genes involved in stress response, secondary metabolism, hormone metabolism, and various other pathways (Figure 5B). Additionally, both KEGG enrichment analyses highlighted numerous pathways related to secondary metabolism (Figure S1).

**Figure 6:**
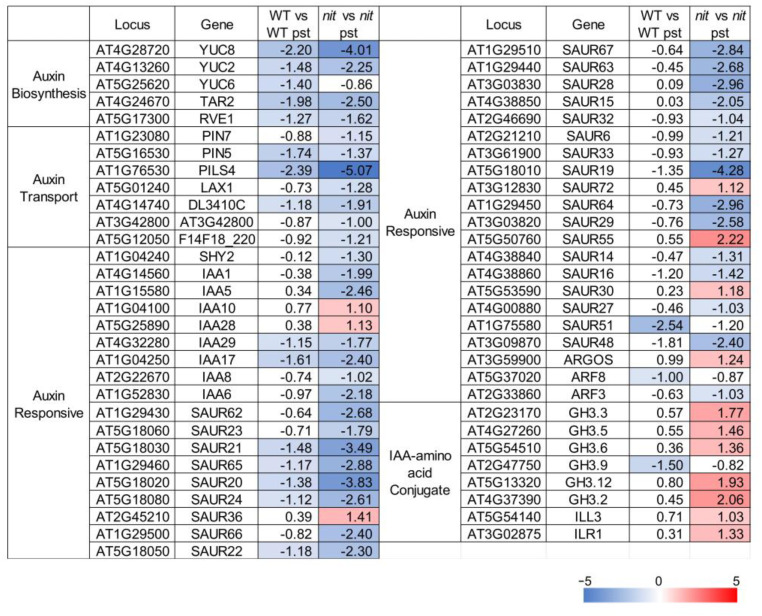
The transcription profile of auxin-related genes. The value represents the Log_2_(Fold Change). Genes that exhibit a Log_2_(Fold Change) ≥ 1 and a *p*-value ≤ 0.05 are color-coded for emphasis. Upregulated genes are highlighted in red, while downregulated genes are highlighted in blue.

The nitrilases NIT1/2/3 are recognized for their role in the conversion of indole-3-acetonitrile (IAN) to the phytohormone indole-3-acetic acid (IAA). Auxin, besides its role in plant growth and development, serves as a pivotal regulator of plant immunity. Hence, we conducted a transcriptome analysis focusing on genes associated with auxin biosynthesis, transport, responsiveness, and IAA–amino acid conjugation (Figure 6). Via clustering, it is evident that the response of the *nit1nit2nit3* to *Pst*DC3000 differs from that of the WT to *Pst*DC3000. After three days of *Pst*DC3000 treatment, the expression of genes related to auxin biosynthesis, transport, and response pathways decreased to different extents in the WT. However, the expression levels of most genes related to auxin biosynthesis and signaling pathways were notably reduced in *nit1nit2nit3* following *Pst*DC3000 treatment, except for a few genes, and the reduction was more pronounced compared to the WT. Conversely, GH3 family genes, which are responsible for facilitating the conjugation of free IAA with amino acids to produce bound IAA, were significantly upregulated in *nit1nit2nit3* after *Pst*DC3000 treatment. However, these findings do not account for the positive regulatory impact of NIT1/2/3 in combating *Pst*DC3000. Hence, it is hypothesized that the resistance conferred by NIT1/2/3 against *Pst*DC3000 may not depend on the auxin pathway.

### 2.5. Enhancement of Arabidopsis Resistance to PstDC3000 by Exogenous 4MSB Is Dependent on NIT1/2/3

**Figure 7:**
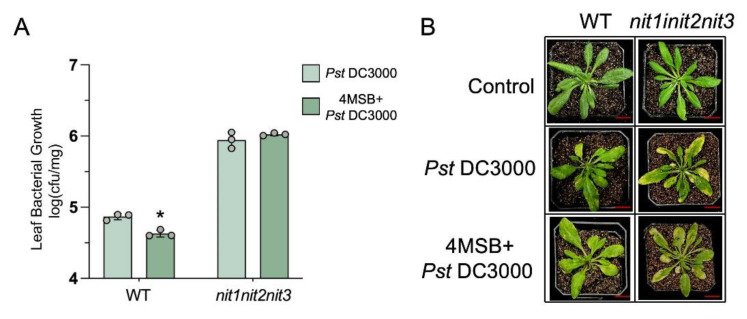
The detection of disease resistance in 28-day-old plants infiltrated with *Pst*DC3000 or 4MSB and *Pst*DC3000. (**A**) Bacterial growth was assessed in leaves of wild-type (WT) and *nit1nit2nit3* plants infected with *Pst*DC3000 or pre-treated with 50 μM 4-methylsulfinylbutyl isothiocyanate (4MSB) and then infected with *Pst*DC3000 at 3 days post-injection (dpi). Each sample consisted of leaves from three plants, with three biological replicates collected for each treatment (dots represent individual biological replicates). The asterisks positioned above the bar represent statistically significant variances between the WT treated with *Pst*DC3000 and the WT treated with *Pst*DC3000 following pre-treatment with 50 μM 4MSB, according to *p* < 0.05 (*) via Student’s *t*-test. (**B**) The 28-day-old plants were subjected to infiltration with either a mock treatment or *Pst*DC3000, or pre-treated with 50 μM 4MSB before being infected with *Pst*DC3000. Subsequently, they were photographed at 3 dpi. Bar = 1 cm.

Sugiyama et al. discovered that upon the exogenous administration of aliphatic glucosinolate 4-methylsulfyl-*n*-butyl glucosinolate (4MSB) to Arabidopsis, nitriles derived from 4MSB were observed in vivo. However, the precise biological role of these compounds remains ambiguous [27]. During *Pst*DC3000 infection, we externally administered 4MSB to both the WT and *nit1nit2nit3*. Our findings revealed that 4MSB application enhanced the resistance of the WT to *Pst*DC3000, whereas *nit1nit2nit3* did not exhibit a significant change in resistance to the pathogen (Figure 7). These findings imply that the enhancement of *Pst*DC3000 resistance through 4MSB is reliant on the presence of NIT1/2/3.

## 3. Discussion

The disease resistance of plants is influenced by factors including the plant’s genetic background, the pathogen type, and external environmental conditions, as well as the plant’s developmental stage. Arabidopsis demonstrates enhanced *Pst*DC3000 resistance during its mature growth stages compared to its young stage, with distinct disease resistance mechanisms potentially operating in different stages [28,29]. Our study demonstrates that nitrilases NIT1/2/3 positively regulate resistance to *Pst*DC3000 in the mature stage, with no significant impact observed in the young stage. This finding suggests that the disease resistance conferred by NIT1/2/3 is intricately linked to developmental stages, highlighting the multifaceted and varied functions of nitrilases NIT1/2/3.

Auxins play a pivotal role in mediating the intricate interactions between plants and pathogens, harmonizing plant growth, development, and defense mechanisms. The induction of the auxin signaling pathway facilitates the entry of biotrophic and hemibiotrophic pathogens into plants. Notably, the innocuous effector protein AvrRpt2, secreted by *Pseudomonas syringae*, infiltrates plant cells, stimulating auxin production, which exerts a suppressive influence on the resistance against *Pst*DC3000 [19]. In our study, we noted a rise in auxin levels following treatment with *Pst*DC3000 (Figure 2). However, RNA-seq analyses showed that the expression of most auxin pathway-related genes in the WT and *nit1nit2nit3* decreased after three days of treatment with *Pst*DC3000, with a greater decrease observed in *nit1nit2nit3*. Notably, the expression of *GH3* family genes was upregulated (Figure 6). GH3 family genes serve as bifunctional modulators in SA and auxin signaling during plant disease resistance [30]. However, further investigation is needed to determine whether NIT1/2/3 regulate plant disease resistance through the GH3 family. On the other hand, auxin distribution in *nit1*, *nit2*, and *nit3* is uneven at the mature stage but becomes evenly distributed, similar to that of the WT, after exposure to *Pst*DC3000 treatment (Figure 2). However, further research is required to ascertain whether the transport and distribution of auxin in *nit1/2/3*-deficient mutants has a direct or indirect impact on plant disease resistance.

**Figure 8:**
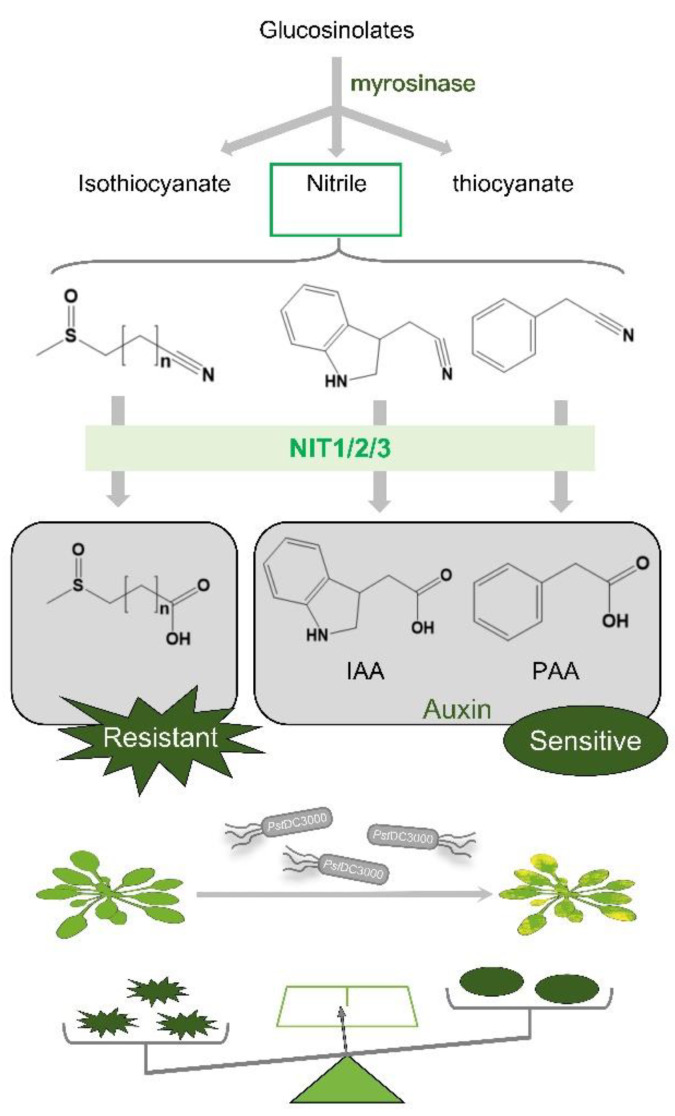
A model of the effect of NIT1/2/3 on *Pst*DC3000 resistance in Arabidopsis.

Indole glucosinolates have been shown to degrade into indole-3-acetonitrile (IAN), which is subsequently converted to IAA through the catalytic activity of NIT1/2/3. Similarly, aromatic glucosinolate degradation products can also undergo NIT1/2/3-mediated conversion to phenylacetic acid (PAA). Nevertheless, the enzymatic conversion of aliphatic glucosinolate degradation products by NIT1/2/3 into their corresponding carboxylic acid derivatives remains unexplored [26,31]. The substrate versatility of nitrilases suggests their potential to degrade nitriles formed during the breakdown of aliphatic glucosinolates, potentially generating compounds resistant to *Pst*DC3000. NIT1/2/3 likely play dual roles in disease resistance and defense mechanisms. Following *Pst*DC3000 infection, NIT1/2/3 facilitate auxin biosynthesis, triggering certain disease responses. Simultaneously, these enzymes break down nitrile compounds derived from aliphatic glucosinolates, contributing to disease resistance. It is hypothesized that upon Arabidopsis infection with *Pst*DC3000, NIT1/2/3 preferentially catalyze aliphatic glucosinolates to bolster plant immunity against pathogens (Figure 8).

## 4. Materials and Methods

### 4.1. Plant Material and Growth Conditions

The following, previously described T-DNA insertion, transgenic, and hybrid lines were used: *nit1* (Salk_114153), *nit2* (Salk_207800), *nit3* (CS324250), *ProNIT1::GUS*, *ProNIT2::GUS*, *ProNIT3::GUS*, *ProDR5::GUS nit1*, *ProDR5::GUS nit2*, and *ProDR5::GUS nit3* [26]. All these lines and the wild type (WT) were of the Columbia-0 (Col-0) ecotype. *nit1nit2nit3* was generated through the targeted disruption of *NIT1* and *NIT3* in the *nit2* line employing CRISPR/Cas9 technology (Figure S2). The sgRNA sequences for *NIT1* (GAGCAAGACTAGTGTTGTTCC) and *NIT3* (GTAACTCGTACGGTGGAGGA) were designed using the Crispr online tool [32]. The *pBE1.1-zmpl Cas9* vector served as the template for assembling the entire sgRNA expression cassette. The sgRNA expression cassette was integrated into the *pBE1.1bdcas9i* vector. Following *Agrobacterium tumefaciens*-mediated transformation, transformants were subjected to kanamycin screening, ultimately leading to the identification of the homozygous *nit1nit2nit3* line through sequencing.

For seedlings cultured in 1/2MS medium (with 3% sucrose, pH = 5.8), seeds were surface sterilized with a 70% ethanol solution for 30 s and subsequently sterilized with seed disinfectant (2% PPM, 0.1% TritonX-100) for 4–8 h. The seed disinfectant was replaced and vernalization was carried out at 4 °C for 3 days. For seedlings cultured in soil, the seeds were vernalized directly at 4 °C for 3 days. All the plants were cultivated under a regular condition, with a 16 h light and 8 h dark photoperiod, a light intensity of 100 µmol•m^−2^ •s^−1^, a temperature of 23 °C, and a relative humidity of 60%.

### 4.2. The GUS Staining

Seedlings were GUS-stained using a previously described histochemical method [33]. At least 10 plants were observed for each sample. The samples were examined and documented using a stereo microscope, with photographs depicting the most illustrative results.

### 4.3. Bacterial Inoculation and Quantitative Assays

*Pseudomomas syringae* pv. *tomato* DC3000 (*Pst*DC3000) was cultured on King’s B (KB) medium at 28 °C for 48 h. The colonies were shaken in liquid KB medium containing 100 μg mL^−1^ rifampicin and 50 μg mL^−1^ kanamycin for 12 h. After centrifugation, cells were diluted to 10^8^ colony-forming units (CFU) mL^−1^ in 10 mM MgCl_2_ (supplemented with 0.04% Silwet L-77). The bacterial suspension was sprayed onto the surface of the rosettes. Control plants were mock inoculated with 10 mM MgCl_2_ (supplemented with 0.04% Silwet L-77). The plants were covered with plastic wrap to maintain high humidity, and bacterial growth was monitored at 3 days (16 h light/8 h dark) after continuous inoculation. The fresh weight of each group of samples was recorded and the rosettes’ surface was disinfected with 70% ethanol. The rosettes were ground with ddH_2_O to homogenate them. Serial dilutions were plated on KB medium containing 100 μg mL^−1^ rifampicin and 50 μg mL^−1^ kanamycin for bacterial colonization statistics.

### 4.4. Cell Death Measurement

Cell death was evaluated using a trypan blue labeling assay. The rosettes were completely immersed in the trypan blue solution (0.4%, Biotopped) and kept away from light for 30 min at room temperature. They were decolorized overnight with 98% ethanol. The fully decolorized and representative samples were selected and photographed.

### 4.5. RNA Sequencing Analysis

Four-week-old WT and *nit1nit2nit3* seedlings were treated with *Pst*DC3000 and 10 mM MgCl_2_ (supplemented with 0.04% Silwet L-77) for a duration of three days; three independent biological replicates were selected from each experimental group for RNA sequencing (RNA-Seq). Total RNA was extracted with the Ultrapure RNA Kit (Cwbio), with subsequent cDNA library construction and sequencing services provided by Applied Protein Technology Biotechnology Co., Ltd. (Shanghai, China) utilizing the Illumina NovaSeq6000 sequencing platform. Differential expression analysis between groups was conducted with DESeq2 (http://bioconductor.org/packages/release/bioc/html/DESeq2.html, accessed on 8 August 2025), using a significance threshold of *p*-value < 0.05 and an absolute Log_2_(FoldChange) value > 1 to screen for differentially expressed genes (DEGs). The Arabidopsis TAIR10 genome served as the reference genome. DEGs were subjected to visual enrichment analysis through Mapman software (Version 3.7.0) [34]. The GO functional enrichment and KEGG pathway enrichment analyses were conducted using the clusterProfiler R software package (Version 1.46.0).

## Data Availability

The raw sequencing data of all samples have been uploaded to the SRA database with the accession number PRJNA1145371 (http://www.ncbi.nlm.nih.gov/bioproject/1145371, accessed on 8 August 2025).

## Supplementary Materials

The following supporting information can be downloaded at https://www.mdpi.com/article/10.3390/ijms252312895/s1.
