# Supplementary material for: Nitrilases NIT1/2/3 Positively Regulate Resistance to Pseudomonas syringae pv. tomato DC3000 Through Glucosinolate Metabolism in Arabidopsis
Source: Int J Mol Sci. 2024 Nov 30;25(23):12895. doi: 10.3390/ijms252312895 (PMC11641764; doi:10.3390/ijms252312895)
Supplement: Supplementary file 1 [file ijms-25-12895-s001.zip › ijms-3309669-supplementary.pdf]

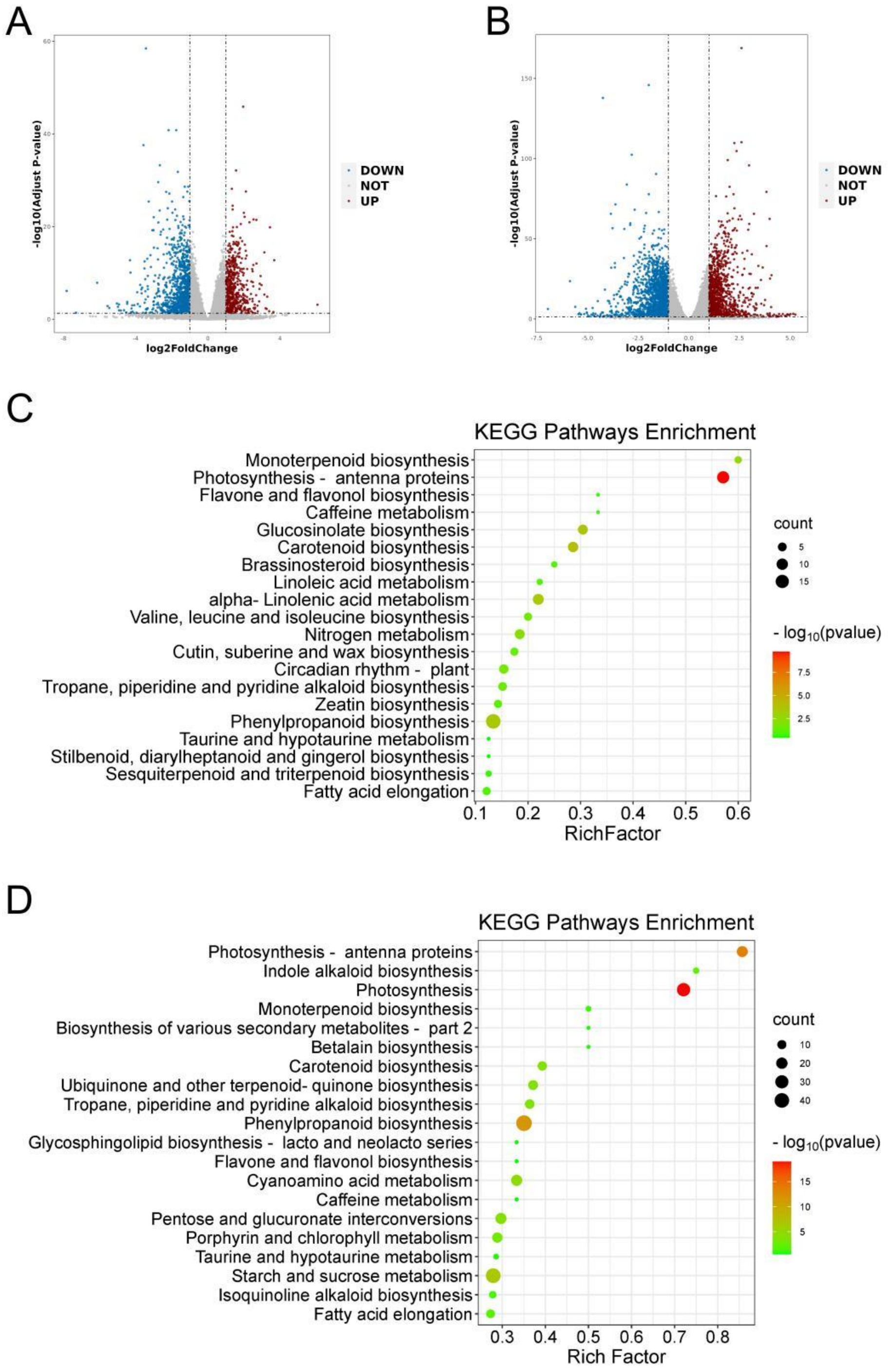

Figure S1. Transcription profile data analysis of hub genes.

A. WT vs WT- *Pst*DC3000 differential gene volcano map.

B. *nit1nit2nit3* vs *nit1nit2nit3-Pst*DC3000 differential gene volcano map.

C. KEGG enrichment analysis of differentially expressed genes of WT vs WT-*Pst*DC3000.

D. KEGG enrichment analysis of differentially expressed genes of *nit1nit2nit3* vs *nit1nit2nit3-Pst* DC3000

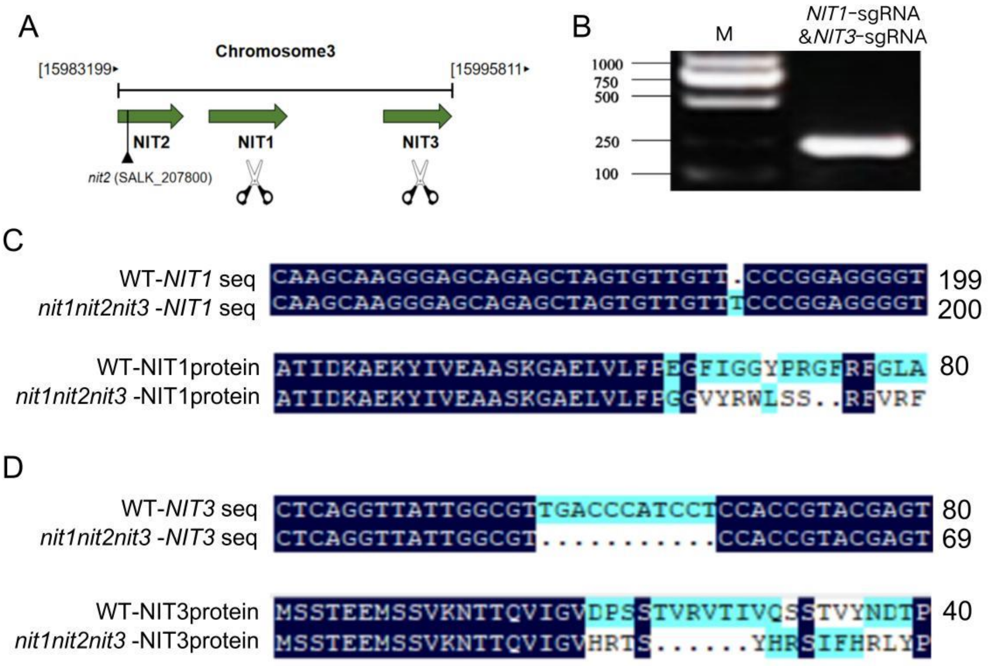

Figure S2. Construction of *nit1nit2nit3* three deletion mutant.

A. Schematic construction diagram of *nit1nit2nit3* triple-deletion mutant using CRISPR/Cas9.

B. Construction of *NIT1*-sgRNA & *NIT3*-sgRNA expression cassette.

C. Alignment of *nit1nit2nit3* deletion mutants and WT on NIT1 nucleotide sequences and amino acid sequences.

D. Alignment of *nit1nit2nit3* deletion mutants and WT on NIT3 nucleotide sequences and amino acid sequences.
